# Metagenome-assembled genomes from methane-fed bottle incubations under variable moisture and pH conditions

**DOI:** 10.1128/mra.01041-26

**Published:** 2026-10-15

**Authors:** Camila Gonzalez, Johannes Ali, Verónica Román-Reyna, Juliana Vasco-Correa

**Affiliations:** 1 Department of Agricultural and Biological Engineering, The Pennsylvania State University 8082, University Park, Pennsylvania, USA; 2 Department of Plant Pathology and Environmental Microbiology, The Pennsylvania State University 8082, University Park, Pennsylvania, USA; 3 One Health Microbiome Center, The Pennsylvania State University 8082, University Park, Pennsylvania, USA; Montana State University, Bozeman, Montana, USA

**Keywords:** methanotrophs, methane oxidation, metagenome-assembled genomes, biofiltration, compost microbiome, shotgun metagenomics

## Abstract

Methane-oxidizing microorganisms mitigate methane emissions in natural and engineered environments. We report 63 metagenome-assembled genomes recovered from methane-fed compost incubations under contrasting moisture and pH conditions, including 62 bacterial metagenome-assembled genomes (MAGs) and 1 archaeal MAG. The MAGs include methanotrophs and microorganisms involved in complementary carbon, nitrogen, sulfur, and carbohydrate metabolic functions.

## ANNOUNCEMENT

**Fig 1:**
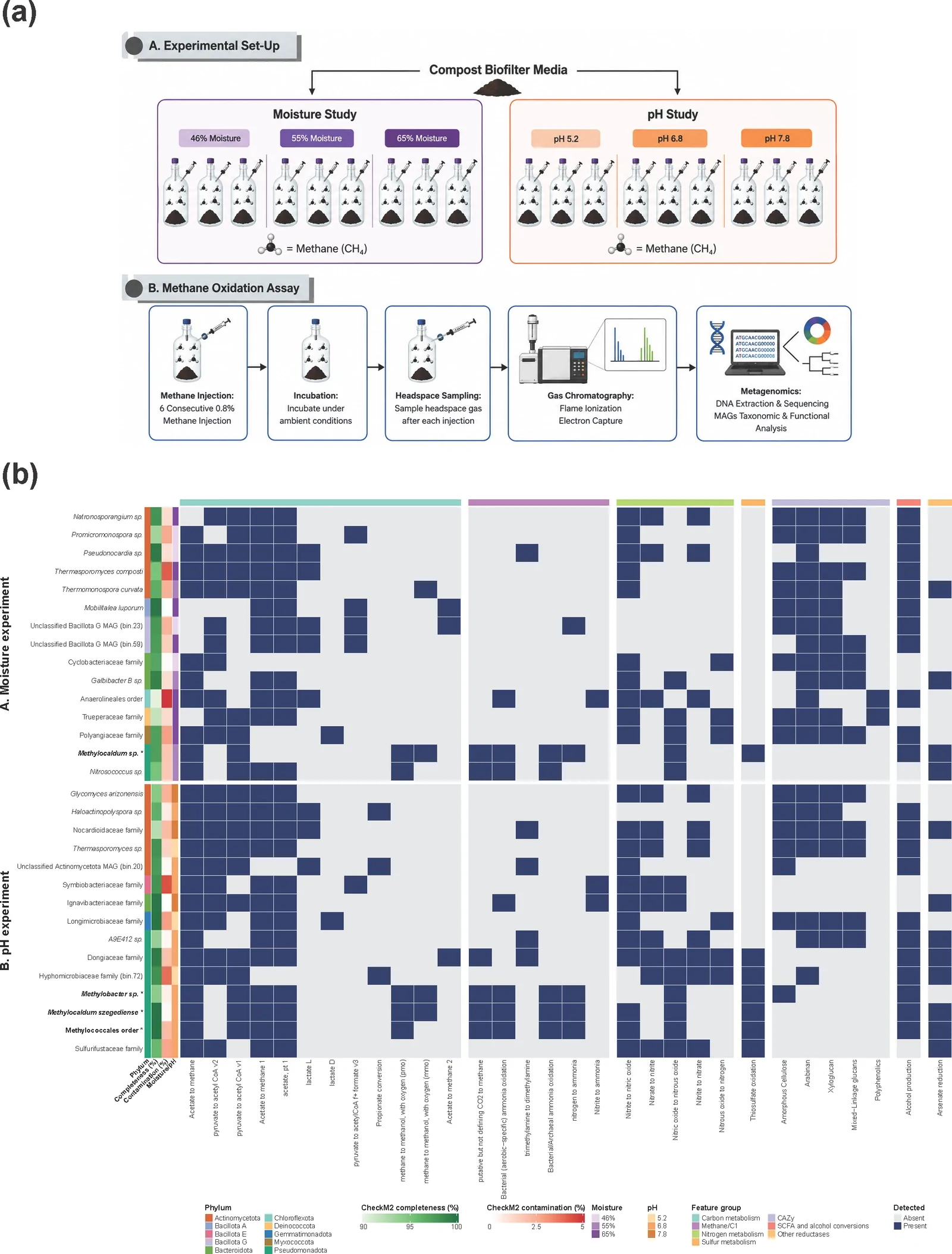
Experimental workflow and metabolic profiles of methane-fed compost bottle incubations. (**a**) Moisture and pH experimental design and methane oxidation assay. (**b**) Selected metabolic genes across 30 representative high-quality MAGs; methanotroph-associated MAGs are shown in bold with a star.

Methane (CH_4_) is a potent greenhouse gas, and compost-based biofiltration can mitigate dilute CH_4_ emissions by supporting methane-oxidizing microbial communities (1). We present a metagenomic resource from CH_4_-fed compost incubations designed to enrich methane-oxidizing communities under contrasting moisture and pH conditions (Fig. 1a).

Compost from the Organic Materials Processing Center at The Pennsylvania State University served as inoculum. Two aerobic experiments with ambient-air headspace evaluated moisture (46%, 55%, and 65%, wet basis) and pH (5.2, 6.8, and 7.8). In the moisture experiment, native compost remained at 46% moisture, whereas the 55% and 65% treatments were adjusted with ultrapure water and pH remained 6.8. In the pH experiment, all treatments were adjusted to 55% moisture, and pH was adjusted with 0.1 M phosphate buffer. Each treatment consisted of three biological replicates, each containing compost equivalent to 15 g dry mass. Bottles were prepared aerobically with a 1.243 L ambient-air headspace. Bottles were incubated statically at 35°C and received six consecutive 10 mL pure CH_4_ amendments (approximately 0.8% vol/vol CH_4_). Methane was monitored using an SRI 8610C gas chromatograph equipped with a methanizer and flame ionization detector. Methane was re-amended when its concentration was undetectable. Cycles lasted 8–136 h, and assays ended after six cycles. Following the final cycle, compost was collected for metagenomic sequencing. A 0.25 g aliquot from each biological replicate was processed independently for DNA extraction. Total DNA was extracted using the DNeasy PowerSoil Pro Kit (Qiagen). Samples were stored at −80°C; DNA concentration was measured using a Qubit double-stranded DNA assay, and DNA integrity was assessed using 0.7% agarose gel electrophoresis. Initial compost collected before methane exposure served as a baseline.

Shotgun metagenomic libraries were prepared by Novogene Corporation (Sacramento, CA, USA) using the Library DNA Prep Kit (Illumina) and sequenced on an Illumina NovaSeq X Plus platform using 2 × 150 bp paired-end sequencing. Sequencing generated 53.7–97.5 million raw reads per sample (8.1–14.6 Gb). Default parameters were used except where noted. Reads were quality filtered using fastp v1.3.3 (2), assembled with metaSPAdes v4.2.0 (3), and evaluated using QUAST v5.3.0 (4). Quality-filtered reads were mapped using BWA v0.7.17 (5) and SAMtools v1.18 (6). Metagenome-assembled genomes (MAGs) were reconstructed using MetaBAT2 v2.17.77 (7), refined with DAS Tool v1.1.7 (8), and assessed using CheckM2 v1.0.2 (9). MAGs with ≥90% completeness and ≤5% contamination were retained as high quality. High-quality MAGs were dereplicated with dRep v3.6.2 (10) at 95% average nucleotide identity, retaining one representative per cluster. Representative MAGs were classified using GTDB-Tk v2.4.0 (11), quantified using CoverM v0.7.0 (12), and functionally annotated with DRAM v1.5.0 (13).

Analyses recovered 63 MAGs, including 62 bacterial MAGs representing 12 bacterial phyla and 1 archaeal MAG (Table 1). Genome quality, assembly statistics, coverage, GenBank WGS accessions, and GTDB-Tk assignments are summarized in Table 1. DRAM annotation identified methanotrophs and populations involved in carbon, nitrogen, sulfur, and carbohydrate metabolism (Fig. 1b). Pathway completeness profiles are available in Figshare at https://doi.org/10.6084/m9.figshare.33235683. This data set provides a genome-resolved resource for studies of methane-oxidizing compost communities and engineered methane biofiltration systems.

**TABLE 1:** Genome characteristics and taxonomic assignments of the MAGs recovered from methane-fed compost bottle incubations^*a*^

MAG ID	Treatment	GenBank WGS accession	Completeness (%)	Contamination (%)	Genome size (Mbp)	Contigs	N50 (bp)	GC (%)	Predicted coding DNA sequences	Coverage (×)	GTDB-Tk classification
E41-L5.bin.23	Moisture 46%	JCBQPT000000000	97.82	2.36	3.019	123	44,062	44.94	2,706	12.18	UBA8346 family
E41-L5.bin.48	Moisture 46%	JCBQPS000000000	93.39	0.99	3.904	295	17,500	52.36	3,379	9.79	*Anseongella* sp.
E41-L5.bin.60	Moisture 46%	JCBQPR000000000	99.80	0.29	2.575	35	101,431	48.16	2,279	13.00	*Edaphocola* sp.
E41-L5.bin.61	Moisture 46%	JCBQPQ000000000	100.00	0.16	3.335	160	39,770	75.51	3,201	29.42	ZC4RG19 sp017577565
E42-L5.bin.15	Moisture 46%	JCBQPP000000000	97.69	0.00	3.870	208	28,319	62.94	3,825	21.73	*Paracoccus alkanivorans*
E42-L5.bin.4	Moisture 46%	JCBQPO000000000	98.85	0.35	4.221	36	248,635	50.52	3,531	27.06	*Anseongella* sp.
E42-L5.bin.48	Moisture 46%	JCBQPN000000000	99.53	1.03	7.318	355	31,875	74.24	7,212	17.37	*Pseudonocardia* sp.
E42-L5.bin.61	Moisture 46%	JCBQPM000000000	93.30	2.59	5.108	564	12,816	72.10	4,729	60.36	*Promicromonospora* sp.
E43-L5.bin.16	Moisture 46%	JCBQPL000000000	97.18	0.52	3.248	173	26,953	52.92	2,823	11.64	*Anseongella ginsenosidimutans* A
E43-L5.bin.20	Moisture 46%	JCBQPK000000000	95.93	0.00	4.289	413	14,974	70.00	4,283	61.08	*Nocardioides luteus*
E43-L5.bin.51	Moisture 46%	JCBQPJ000000000	96.89	0.16	4.140	280	20,229	52.57	3,695	9.76	ZC4RG29 sp.
E51-L5.bin.24	Moisture 55%	JCBQPI000000000	98.22	0.23	2.814	112	39,345	41.39	2,480	17.43	*Methanosarcina thermophila*
E51-L5.bin.64	Moisture 55%	JCBQPH000000000	93.18	0.42	3.799	262	19,965	69.00	3,708	11.35	*Saccharopolyspora rectivirgula*
E52-L5.bin.17	Moisture 55%	JCBQPG000000000	98.06	0.05	3.926	353	14,502	46.08	3,209	14.23	*Membranihabitans sp*.
E52-L5.bin.21	Moisture 55%	JCBQPF000000000	96.43	2.42	5.392	309	27,902	71.87	4,979	16.86	*Thermomonospora curvata*
E52-L5.bin.45	Moisture 55%	JCBQPE000000000	91.19	0.02	2.219	57	56,210	49.19	1,986	12.78	Schleiferiaceae family
E52-L5.bin.49	Moisture 55%	JCBQPD000000000	99.11	1.05	3.073	151	37,692	38.26	2,734	12.08	*Galbibacter* B sp.
E52-L5.bin.58	Moisture 55%	JCBQPC000000000	95.87	0.37	2.824	243	16,702	61.44	2,801	31.11	ZC4RG25 sp003242645
E53-L3.bin.56	Moisture 55%	JCBQPB000000000	95.01	1.72	3.335	301	14,430	52.31	3,422	9.67	*Nitrosococcus* sp.
E53-L3.bin.57	Moisture 55%	JCBQPA000000000	97.30	1.83	4.193	509	10,495	58.59	4,112	18.96	*Methylocaldum* sp.
E61-L5.bin.39	Moisture 65%	JCBQOZ000000000	97.28	2.85	6.877	652	14,233	69.14	6,069	9.86	JAEZYZ01 sp023418705
E61-L5.bin.43	Moisture 65%	JCBQOY000000000	95.57	0.19	3.845	129	45,228	65.15	3,395	35.39	UBA3940 sp002385675
E62-L5.bin.29	Moisture 65%	JCBQOX000000000	99.80	0.01	2.309	73	52,781	34.78	2,124	35.35	*Mobilitalea luporum*
E62-L5.bin.4	Moisture 65%	JCBQOW000000000	92.11	0.76	2.282	87	55,351	48.68	2,345	32.71	*Thermoactinomyces intermedius*
E62-L5.bin.43	Moisture 65%	JCBQOV000000000	91.66	1.14	2.785	266	17,657	69.31	2,675	12.45	Trueperaceae family
E63-L5.bin.10	Moisture 65%	JCBQOU000000000	90.13	4.94	4.742	270	27,098	60.10	4,241	10.29	JAAYXE01 sp012516075
E63-L5.bin.103	Moisture 65%	JCBQOT000000000	91.46	0.98	0.734	94	9,469	39.05	798	8.32	Levybacterales order
E63-L5.bin.12	Moisture 65%	JCBQOS000000000	99.97	0.05	2.825	31	134,730	54.00	2,497	15.44	Schleiferiaceae family
E63-L5.bin.3	Moisture 65%	JCBQOR000000000	96.01	0.27	0.673	15	182,384	38.46	716	21.07	UBA1006 sp.
E63-L5.bin.30	Moisture 65%	JCBQOQ000000000	97.86	1.20	3.070	282	14,937	33.59	2,883	11.11	BRH-c54 sp.
E63-L5.bin.54	Moisture 65%	JCBQOP000000000	98.03	1.10	4.370	352	18,514	71.09	4,193	16.88	*Natronosporangium* sp.
E63-L5.bin.59	Moisture 65%	JCBQOO000000000	97.53	1.58	3.517	259	21,568	42.91	3,224	13.78	JAAYHS01 sp.
E63-L5.bin.94	Moisture 65%	JCBQON000000000	95.02	4.21	4.485	250	30,850	69.93	4,055	13.05	*Thermasporomyces composti*
PHC1-L5.bin.14	Initial compost (pH)	JCBQOM000000000	99.99	0.21	4.283	213	32,078	57.15	3,932	15.48	*Methylocaldum szegediense*
PHF1-L4.bin.58	pH 5.2	JCBQOL000000000	99.93	1.55	3.738	228	25,344	68.31	3,388	43.25	*Sphaerobacter thermophilus*
PHF1-L4.bin.59	pH 5.2	JCBQOK000000000	100.00	2.83	4.749	232	39,796	66.81	4,243	12.42	Longimicrobiaceae family
PHF1-L4.bin.6	pH 5.2	JCBQOJ000000000	100.00	0.22	4.354	185	38,741	68.93	4,072	24.97	Thermocrispum municipale
PHF1-L4.bin.72	pH 5.2	JCBQOI000000000	98.20	3.87	4.325	235	28,279	63.35	4,151	20.16	ZC4RG25 sp.
PHF1-L4.bin.74	pH 5.2	JCBQOH000000000	98.12	1.48	4.237	198	35,710	41.04	3,698	11.00	Chitinophagaceae family
PHF2-L4.bin.51	pH 5.2	JCBQOG000000000	98.12	1.39	5.522	137	71,870	67.15	4,835	33.62	*Thermasporomyces* sp.
PHF2-L4.bin.54	pH 5.2	JCBQOF000000000	90.56	2.82	3.738	256	24,053	71.65	3,389	62.96	RSA9 sp.
PHF3-L4.bin.3	pH 5.2	JCBQOE000000000	96.28	1.32	2.770	80	54,838	54.20	2,710	11.41	Micavibrionaceae family
PHS1-L4.bin.39	pH 6.8	JCBQOD000000000	96.36	0.63	4.345	338	18,672	69.01	4,164	12.70	*Haloactinopolyspora* sp021793295
PHS1-L4.bin.47	pH 6.8	JCBQOC000000000	98.13	0.46	3.231	243	20,449	65.97	3,345	13.79	ZC4RG17 sp.
PHS2-L4.bin.33	pH 6.8	JCBQOB000000000	98.13	4.44	3.362	161	33,566	69.31	3,252	20.32	*Symbiobacterium* sp.
PHS2-L4.bin.44	pH 6.8	JCBQOA000000000	93.64	0.30	3.621	84	93,146	64.20	3,468	19.58	A9E412 sp.
PHS3-L5.bin.11	pH 6.8	JCBQNZ000000000	93.74	4.65	4.534	367	18,630	70.52	4,100	13.12	RSA9 family
PHS3-L5.bin.25	pH 6.8	JCBQNY000000000	97.86	0.24	1.945	88	36,115	56.45	1,976	21.24	*Bacillus* BB sp001884825
PHS3-L5.bin.26	pH 6.8	JCBQNX000000000	90.26	0.14	2.173	270	10,127	74.69	1,840	27.38	*Thermaerobacter* sp004634385
PHS3-L5.bin.28	pH 6.8	JCBQNW000000000	94.24	0.05	2.771	90	47,145	39.58	2,651	12.65	*Aequorivita* sp.
PHS3-L5.bin.4	pH 6.8	JCBQNV000000000	95.86	2.68	3.366	228	26,732	70.79	3,227	12.42	JACQFZ01 sp.
PHS3-L5.bin.5	pH 6.8	JCBQNU000000000	100.00	2.10	4.364	295	21,644	59.66	4,009	12.92	Methylococcales order
PHS3-L5.bin.50	pH 6.8	JCBQNT000000000	94.16	0.53	3.096	295	13,068	53.13	2,925	9.71	*Methylobacter* sp.
PHS3-L5.bin.58	pH 6.8	JCBQNS000000000	99.51	1.92	4.820	161	54,969	67.76	4,625	17.40	SYSU-D60014 sp.
PHT1-L5.bin.26	pH 7.8	JCBQNR000000000	99.81	0.02	3.358	126	43,799	33.51	2,993	12.24	RBG-16-34-14 sp.
PHT1-L5.bin.38	pH 7.8	JCBQNQ000000000	94.42	2.39	4.842	367	18,517	70.33	4,630	10.64	*Glycomyces arizonensis*
PHT1-L5.bin.52	pH 7.8	JCBQNP000000000	94.84	0.53	3.029	104	55,882	67.08	3,038	51.23	ZC4RG17 sp.
PHT2-L5.bin.20	pH 7.8	JCBQNO000000000	90.19	1.29	3.484	346	13,626	74.53	3,553	9.82	Egibacteraceae family
PHT2-L5.bin.28	pH 7.8	JCBQNN000000000	98.58	1.03	2.911	283	15,663	70.08	2,747	68.89	CALBZE01 sp.
PHT2-L5.bin.40	pH 7.8	JCBQNM000000000	92.26	2.11	4.103	362	15,329	71.65	4,027	9.53	Nocardioidaceae family
PHW1-L5.bin.20	Water control (pH ~7)	JCBQNL000000000	97.72	0.26	2.592	94	57,295	69.50	2,653	20.68	ZC4RG17 sp017577575
PHW3-L5.bin.47	Water control (pH ~7)	JCBQNK000000000	95.26	3.51	3.461	378	12,241	72.13	3,332	26.38	RSA9 sp.
Pre1-L1.bin.25	Initial compost (moisture)	JCBQNJ000000000	96.70	0.79	3.918	64	126,272	67.65	3,684	22.63	Kaistiaceae family

^*a*^ MAG identifiers encode the source experiment: E denotes the moisture experiment (E4x, 46%; E5x, 55%; E6x, 65%, where x indicates the biological replicate); PH denotes the pH experiment (PHF, pH 5.2; PHS, pH 6.8; PHT, pH 7.8; PHW, unbuffered water control at approximately pH 7; PHC, initial compost); and Pre denotes the initial compost for the moisture experiment.

## Data Availability

This BioProject has been deposited in NCBI under BioProject accession PRJNA1504349. The raw sequencing reads are available in the Sequence Read Archive (SRA) under accessions SRR39904525–SRR39904547. The high-quality nonredundant metagenome-assembled genomes (MAGs) have been deposited in GenBank; individual genome accession numbers are provided in Table 1. The pathway completeness analysis is available on Figshare at https://doi.org/10.6084/m9.figshare.33235683.
